# A Primary Screening Method for Liver Cancer in Chronic Hepatitis B Carriers: A Prospective Community-Based Cohort Study

**DOI:** 10.3389/fonc.2021.762662

**Published:** 2022-01-03

**Authors:** Maomao Cao, He Li, Dianqin Sun, Siyi He, Changfa Xia, Lin Lei, Ji Peng, Wanqing Chen

**Affiliations:** ^1^ Office of Cancer Screening, National Cancer Center/National Clinical Research Center for Cancer/Cancer Hospital, Chinese Academy of Medical Sciences and Peking Union Medical College/Chinese Academy of Medical Sciences Key Laboratory for National Cancer Big Data Analysis and Implement, Beijing, China; ^2^ Department of Cancer Prevention and Control, Shenzhen Center for Chronic Disease Control, Shenzhen, China

**Keywords:** primary liver cancer screening, high-risk population, incidence risk, prediction model, epidemiological risk factors

## Abstract

**Background:**

Patients with hepatitis B virus (HBV) were invited to receive ultrasound and alpha-protein examination directly in China. However, not all HBV carriers need to be subjected to further tests. This study aimed to develop a feasible primary screening method to narrow down potential high-risk individuals of liver cancer among populations with HBV.

**Methods:**

Based on a prospective community-based cohort, potential risk factors were selected as the predictors, including age, sex, smoking, alcohol consumption, diabetes, liver cancer family history, liver diseases in mothers, source of water, body mass index (BMI), and psychological trauma. Cox proportional regression model was applied to predict the 3-year absolute risk of liver cancer and derive risk scores. The area under receiver operating characteristic curve (AUROC) and calibration plot were used to assess the performance of the model. Bootstrap resampling was used for internal validation.

**Results:**

Age, sex, BMI, alcohol consumption, liver diseases in mothers, and psychological trauma were independent risks of liver cancer. The 1- to 3-year AUROC of the prediction model was 71.15% (95% CI, 66.88–75.42), 71.16% (95% CI, 67.42–74.90), and 72.95% (95% CI, 64.20–81.70), respectively. The predicted risk was calibrated well with the observed liver cancer risk. Bootstrap resampling showed that C-index was 0.70 (0.67–0.74). A 32-point risk score was also developed and a score over 5 was identified for patients at extremely high risk.

**Conclusions:**

A user-friendly primary screening method was created that could estimate the 3-year absolute risk of liver cancer and identify extremely high-risk individuals among the population with HBV.

## Introduction

In 2016, World Health Organization (WHO) set an ambitious goal of viral hepatitis elimination by 2030, and reducing the hepatitis B virus (HBV)- and hepatitis C virus (HCV)-associated mortality by 65% (1). As an important cause of HBV-related mortality in China and worldwide, liver cancer represented 21% of mortality globally (2). Thus, to meet the WHO targets, the importance is to reduce the liver cancer burden. Liver cancer screening offers substantial potential to detect cases at an early stage and prolong their survival rate (3–5). In China, liver cancer screening has been covered by three National Key Public Health Services, which provide free screening services aiming at high-risk population or individuals from high-risk regions (6).

Fifty percent to 80% of primary liver cancer cases result from HBV chronic infection (7); thus, hepatitis B surface antigen (HBsAg) test is an essential and crucial method at the primary stage of liver cancer screening to identify high-risk individuals. According to the Technological Program on Cancer Early Detection and Treatment in 2011 in China (8), patients with HBV chronic infection were invited to receive ultrasound and alpha-fetoprotein (AFP) examinations directly. Although seropositive for HBsAg could increase the risk of liver cancer, only 6.2%–9.5% of HBV carriers eventually develop liver cancer if without the occurrence of cirrhosis (9). A risk prediction model for liver cancer based on age, sex, HBeAg, and HBV DNA levels also showed that low-risk HBV-infected individuals had similar cumulative probabilities of developing liver cancer compared with the HBsAg-negative participants, suggesting that HBV carriers at low risk may not need the same surveillance scheme as the high-risk HBV carriers (10). The evidence mentioned above suggests that it may be inappropriate for all patients with HBV chronic infection to be screened. For these reasons, identifying extremely high-risk individuals and optimizing the method of primary screening are necessary among patients chronically infected with HBV for liver cancer screening.

Available prediction models for liver cancer generally included serum biomarkers (e.g., white cell count) in a combination with epidemiological information (11–13). However, these models are relatively complex and not easy to use to identify the potential high-risk individuals at the primary stage of liver cancer screening among HBV carriers. In this study, we aimed to develop a noninvasive risk assessment tool to calculate individualized risk and identify extremely high-risk individuals of liver cancer among populations with HBV chronic infection based on traditional epidemiological risk factors of liver cancer only.

## Materials and Methods

### Study Design and Participants

The community-based cohort study on population with high risk of liver cancer (the CCOP-LC cohort) was initiated in 2017 among residents aged 35–70 years in seven sites (Binhai county, Lingbi county, Mengcheng county, Sheyang county, Shenqiu county, Dancheng county, and Yingdong county), which has been described previously (14). All participants had to take immunochromatographic strip tests for the detection of HBsAg; only the HBsAg-positive patients were enrolled in the final cohort. Patients who received antiviral treatment or were diagnosed with liver cancer or other malignant diseases at initial enrollment were excluded.

The epidemiological research has been registered in Chinese Clinical Trial Registry (identifier: ChiCTR-EOC-17012853). This study was approved by the Ethics Review Committee of the Cancer Hospital, Chinese Academy and Medical Sciences. Written informed consent was obtained from all participants.

### Liver Cancer Ascertainment and Follow-Up

At baseline, patients who received liver cancer screening and showed suspicious or positive results were invited to CT/MRI to confirm the diagnosis. A mixed follow-up method was applied at the follow-up stage. An active follow-up approach was conducted initially, and then we linked all cohort populations with the local Cancer Registration database and Death Surveillance database to obtain information on cancer incidence and all-cause mortality, and then cross-referred them to medical insurance databases or medical records from local hospitals. All participants were followed up until December 31, 2020.

### Risk Factors Measurement

A standardized self-administered epidemiological questionnaire was offered to each participant by face-to-face interview to collect demographic characteristics, lifestyle, and medical history. Potential risk factors were selected as the predictors, including age, sex, smoking, alcohol consumption, diabetes, family history of liver cancer, liver diseases in mothers, source of water, body mass index (BMI), and psychological trauma. Candidate risk factors identified for inclusion in this study were those previously shown significantly associated with the development of liver cancer (15, 16). Participants were asked for information on the frequency of alcohol consumption, average alcohol consumption per week, and types of drinks. Ethanol content differed for each type of alcohol and was assumed to be the same measurement method as follows: 180 ml sake (rice wine) as 23 g ethanol, 180 ml white spirits as 36 g, 633 ml beer as 23 g, and 60 ml wine as 6 g (17). Weekly alcohol consumption levels were classified into three groups (never, 0–550 g/ethanol, ≥550 g/ethanol). The question on smoking habits includes current and former smoking status, age at initiation of smoking, average number of cigarettes per day, and types of smoking [1 cigarette = 1 g of tobacco = 0.5 cigars (18)]. The smoking intensity was evaluated by pack-year defined by multiplying the number of years of smoking by the average number of cigarettes per day divided by 20 (19). We classified smokers by the following categories of smoking intensity: never, light (<20 pack-years for females, <30 pack-years for males), and heavy (≥20 pack-years for females and ≥30 for males). BMI was calculated as weight in kilograms divided by height in meters squared and classified into <25 kg/m^2^ and ≥25 kg/m^2^ according to the criteria in the diagnosis of obesity from WHO for Asian and South Asian population (20). History of diabetes was collected through self-report. Perinatal transmission (from mother to infant at birth) is the most common route for the transmission of hepatitis B virus in China, and thus maternal liver disease is an important risk factor for liver cancer. In this study, maternal liver diseases referred to liver diseases in mothers, including liver cancer, chronic hepatitis B, and other liver diseases, which were detected among 1,553 persons, of them, 641 (41.27%) mothers were diagnosed with chronic hepatitis B and 482 (31.04%) mothers had liver cancer.

### Statistical Analysis

Continuous variables were expressed as means ± standard deviations, whereas categorical variables were presented as numbers and percentages. Person-years at risk were counted from enrollment until liver cancer diagnosis, death, or December 31, 2020, whichever occurred first. Pearson’s Chi-square or Fisher exact tests were performed to compare the categorical variables. Cox proportional regression model with the backward method was applied to screen possible independent risk factors and acquire hazard ratios (HRs) and corresponding 95% confidence intervals (CIs). Proportional hazard assumptions were assessed by martingale residual. The time-dependent area under receiver operating characteristic curve (AUROC) and calibration plot were used to assess the discriminatory ability and goodness of fit. A total of 1,000 bootstrap samples were used for internal validation, presenting as concordance index (C-index).

The 3-year absolute risks of liver cancer were calculated using the following standard equation (21, 22):


$$F(t)=1–S{\left(t\right)}^{\exp(f[x,{\text{M}}_{i}])};f[x,M_{i}]=\sum_{i=1}^{P}\beta_{i}X_{i}\sum_{i=1}^{P}\beta_{i}{\bar{x}}_{i}$$

where *F(t)* refers to the probability of developing liver cancer in *t* years; *S(t)* is the baseline disease-free probability; *P* is the number of the statistical variables; *β_i_* is the regression coefficient for the *i*th variables; and ${\bar{x}}_{i}$ is the mean level of *i*th covariates.

A simple-to-use risk score was derived based on the following six steps (21):

1. Selecting independent risk factors and corresponding coefficients obtained from the Cox proportional hazards regression model;
2. Calculating means (or proportions) for each risk-factor category of the risk factors and baseline disease-free probability;
3. Determining a reference value for each category of each risk factor and choosing a base category for each risk factor;
4. Computing weighted distance between each category of each risk factor and base category using *β*_i_×(W_ij_ − W_iREF_);
5. Setting the constant B, and in this study, B = 5*β_age_; and
6. Integer risk score = [*β*_i_×(W_ij_ − W_iREF_)]/B.

To ease the application of the risk score, cutoff values were determined by the Youden index, sensitivity, specificity, and the purpose of the study comprehensively. The 3-year cumulative risk of liver cancer was calculated using the Kaplan–Meier method and compared with the log-rank rest. All statistical tests were two-sided and a *p* of <0.05 was considered statistically significant. Missing data were excluded directly. Analyses were conducted by using SAS version 9.4 (SAS Institute, Cary, NC, USA) and R version 4.0.4.

## Results

### Baseline Characteristics of the Participants

A total of 10,536 patients were included in the present study. At a median follow-up period of 2.64 years, 203 patients developed liver cancer (1.93%). **Table 1** shows the baseline demographic characteristics and liver cancer potential risk factors. Patients diagnosed with liver cancer were more likely to be older (*p* < 0.001). The number of liver cancer cases in males was significantly higher than that in females, accounting for 66.01% and 33.99% of all cases, respectively (*p* < 0.001). BMI, smoking, alcohol consumption, psychological trauma, and liver diseases in mothers also distributed statistically significantly different among patients with liver cancer and without liver cancer (*p* < 0.05).

**Table 1:** Baseline characteristics of the study patients with chronic HBV.

Variables	All (*N* = 10,536)	Liver cancer development (%)		p
		No (*N* = 10,333)	Yes (*N* = 203)	
Age, years				<.001
35–39	550 (5.22)	547 (5.29)	3 (1.48)	
40–44	840 (7.97)	830 (8.03)	10 (4.93)	
45–49	1,615 (15.33)	1,590 (15.39)	25 (12.32)	
50–54	2,059 (19.54)	2,036 (19.70)	23 (11.33)	
55–59	1,627 (15.44)	1,600 (15.48)	27 (13.30)	
60–64	1,874 (17.79)	1,830 (17.71)	44 (21.67)	
65–70	1,971 (18.71)	1,900 (18.39)	71 (34.98)	
Sex				<.001
Male	4,991 (47.37)	4,857 (47.00)	134 (66.01)	
Female	5,545 (52.63)	5,476 (53.00)	69 (33.99)	
Marriage				0.012
Unmarried	167 (1.59)	158 (1.53)	9 (4.43)	
Married	9,649 (91.58)	9,468 (91.63)	181 (89.16)	
Divorced	75 (0.71)	74 (0.72)	1 (0.49)	
Widow	645 (6.12)	633 (6.13)	12 (5.91)	
Educational level				0.486
No schooling	3,462 (32.86)	3,388 (32.79)	74 (36.45)	
Primary school	3,194 (30.32)	3,142 (30.41)	52 (25.62)	
Middle school	3,034 (28.8)	2,973 (28.77)	61 (30.05)	
Junior college above	846 (8.03)	830 (8.03)	16 (7.88)	
Water source				0.283
Cellar water, pond water and shallow well water, Lakes and rivers	2,020 (19.17)	1,973 (19.09)	47 (23.15)	
Deep well water and springs	2,049 (19.45)	2,008 (19.43)	41 (20.20)	
Tap water	6,467 (61.38)	6,352 (61.47)	115 (56.65)	
BMI (kg/m^2^)				0.001
<25	5,461 (51.83)	5,333 (51.61)	128 (63.05)	
≥25	5,075 (48.17)	5,000 (48.39)	75 (36.95)	
Smoking				0.001
Never	7,424 (70.46)	7,306 (70.71)	118 (58.13)	
Light	1,767 (16.77)	1,718 (16.63)	49 (24.14)	
Heavy	1,345 (12.77)	1,309 (12.67)	36 (17.73)	
Alcohol consumption (g/week ethanol)				0.010
No	8,436 (80.07)	8,276 (80.09)	160 (78.82)	
<550	1,949 (18.50)	1,914 (18.52)	35 (17.24)	
≥550	151 (1.43)	143 (1.38)	8 (3.94)	
Psychological trauma				0.005
No	8,080 (76.69)	7941 (76.85)	139 (68.47)	
Yes	2,456 (23.31)	2,392 (23.15)	64 (31.53)	
Liver diseases in mothers				0.027
No	8,983 (85.26)	8,821 (85.37)	162 (79.80)	
Yes	1,553 (14.74)	1,512 (14.63)	41 (20.20)	
Liver cancer family history				0.701
No	8,874 (84.23)	8,705 (84.24)	169 (83.25)	
Yes	1,662 (15.77)	1,628 (15.76)	34 (16.75)	
Diabetes				0.416
No	9,805 (93.06)	9,619 (93.09)	186 (91.63)	
Yes	731 (6.94)	714 (6.91)	17 (8.37)	

### Predictors of the Development of Liver Cancer

The results of univariate and multivariate analysis are summarized in **Table 2**. In the univariate analysis, age (year; HR = 1.05, 95% CI = 1.04–1.07), sex (male; HR = 2.17, 95% CI = 1.62–2.90), BMI (<25; HR = 1.59, 95% CI = 1.20–2.12), smoking (light; HR = 1.76, 95% CI = 1.26–2.46; heavy; HR = 1.68, 95% CI = 1.16-2.45), alcohol consumption (≥550 g/week ethanol; HR = 2.85, 95% CI = 1.40–5.81), liver diseases in mothers (yes, HR = 1.47, 95% CI = 1.05–2.07), and psychological trauma (yes; HR = 1.53; 95% CI = 1.14–2.05) were associated with the development of liver cancer. By multivariate comparison, except for smoking, the following 5 variables were still independently related to the presence of liver cancer: age in years (HR = 1.06, 95% CI = 1.04–1.08), male gender (HR = 2.41, 95% CI = 1.76–3.29), BMI <25 (HR = 1.46, 95% CI = 1.10–1.95), alcohol consumption ≥ 550 g/week ethanol (HR = 2.12, 95% CI = 1.03–4.38), liver diseases in mothers (HR = 1.84, 95% CI = 1.30–2.61), and presence of psychological trauma (HR = 1.62, 95% CI = 1.20–2.18).

**Table 2:** Variables associated with liver cancer development in Cox’s model.

Variables	Pearson-years	Cases	Crude HR(95% CI)	*β* coefficient	Adjusted HR (95% CI)^a^
Age, years	26,769.33	203	1.05 (1.04–1.07)	0.058	1.06 (1.04–1.08)
Sex					
Female	12,614.39	69	ref		ref
Male	14,154.94	134	2.17 (1.62–2.90)	0.879	2.41 (1.76–3.29)
BMI (kg/m^2^)					
≥25	12,913.46	75	ref		ref
<25	13,855.86	128	1.59 (1.20–2.12)	0.38	1.46 (1.10–1.95)
Smoking					
Never	18,900.00	118	ref		
Light	44,34.90	49	1.76 (1.26–2.46)		
Heavy	34,34.42	36	1.68 (1.16–2.45)		
Alcohol consumption (g/week ethanol)					
No	21,442.92	160	ref		ref
<550	4,952.74	35	0.95 (0.66–1.37)	–0.368	0.69 (0.47–1.02)
≥550	373.66	8	2.85 (1.40–5.81)	0.753	2.12 (1.03–4.38)
Liver diseases in mothers					
No	22,859.53	162	ref		ref
Yes	3,909.79	41	1.47 (1.05–2.07)	0.608	1.84 (1.30–2.61)
Liver cancer family history					
No	22,537.16	169	ref		
Yes	4,232.16	34	1.07 (0.74–1.55)		
Diabetes					
No	24,923.28	186	ref		
Yes	18,46.05	17	0.81 (0.49–1.33)		
Water source					
Cellar water, pond water and shallow well water, Lakes and rivers	5,029.67	47	1.33 (0.95–1.87)		
Deep well water and springs	4,985.55	41	1.15 (0.81–1.65)		
Tap water	16,754.11	115	ref		
Psychological trauma					
No	20,582.11	139	ref		ref
Yes	6,187.21	64	1.53 (1.14–2.05)	0.48	1.62 (1.20–2.18)

HR, hazard ratio; CI, confidence interval; ref: reference; ^a^Adjusted by age, sex, BMI, smoking, alcohol consumption, liver diseases in mothers, psychological trauma, marriage status, and educational level.

### Development of the Risk Prediction Model and Derivation of the Risk Score for Liver Cancer

**Figure 1:**
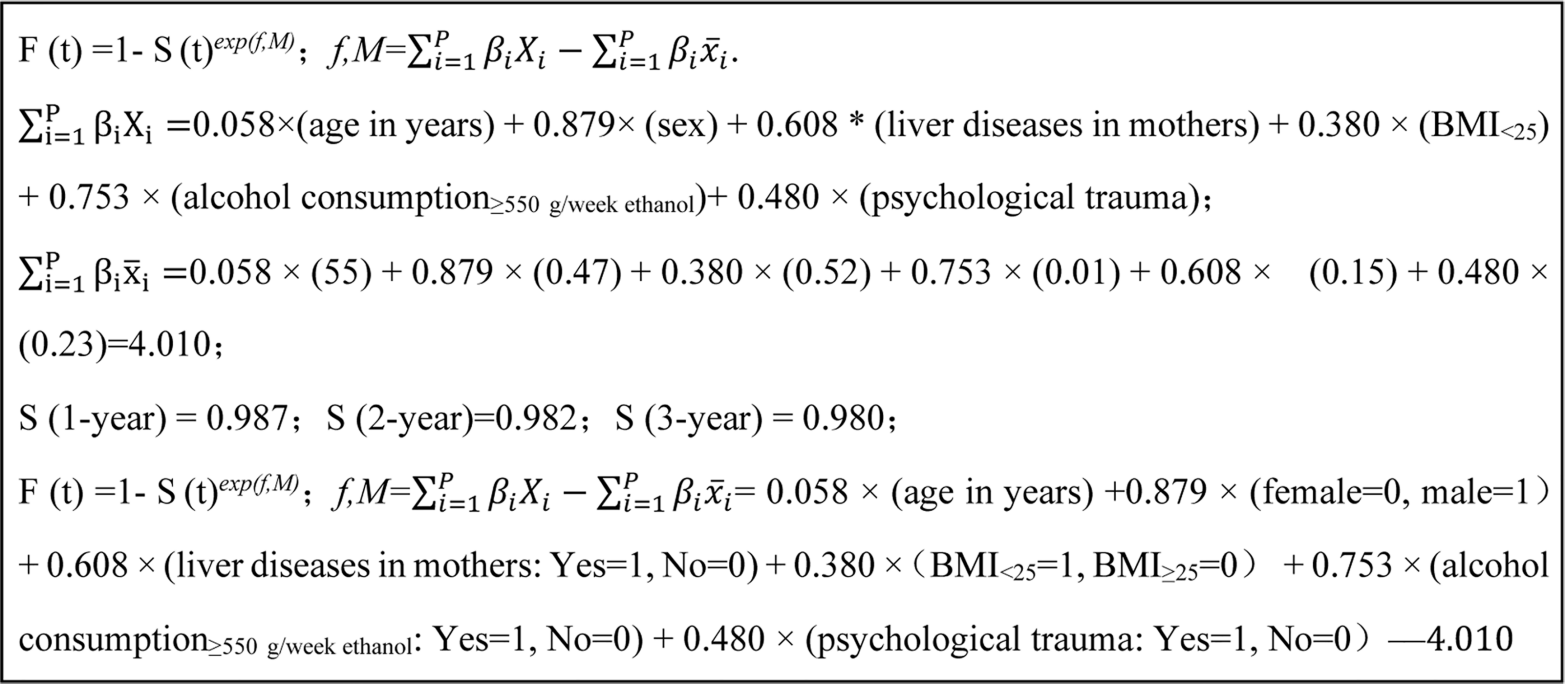
The process and final formula of liver cancer risk prediction model in hepatitis B patients.

The statistically significant variables were selected to construct the risk prediction model, which was presented as follows: *F*(*t*) =1 - *S*(*t*)*^exp(f,M)^*; f,M *=* $\sum_{i=1}^{p}\beta_{i}X_{i}-\sum_{i=1}^{p}\beta_{i}{\bar{x}}_{i}$ = 0.058 × (age in years) + 0.879 × (Female = 0, Male = 1)+ 0.608 × (liver diseases in mother: Yes = 1, No = 0) + 0.380 ×(BMI_<25_ = 1, BMI_≥25_ = 0) + 0.753 × (alcohol consumption_≥550 g/week ethanol_: Yes = 1, No = 0) + 0.480 × (psychological trauma: Yes = 1, No = 0) – 4.010. A detailed process to develop the risk prediction model is shown in **Figure 1**. Individual risk of developing liver cancer can be estimated by using this calculator.

**Figure 2:**
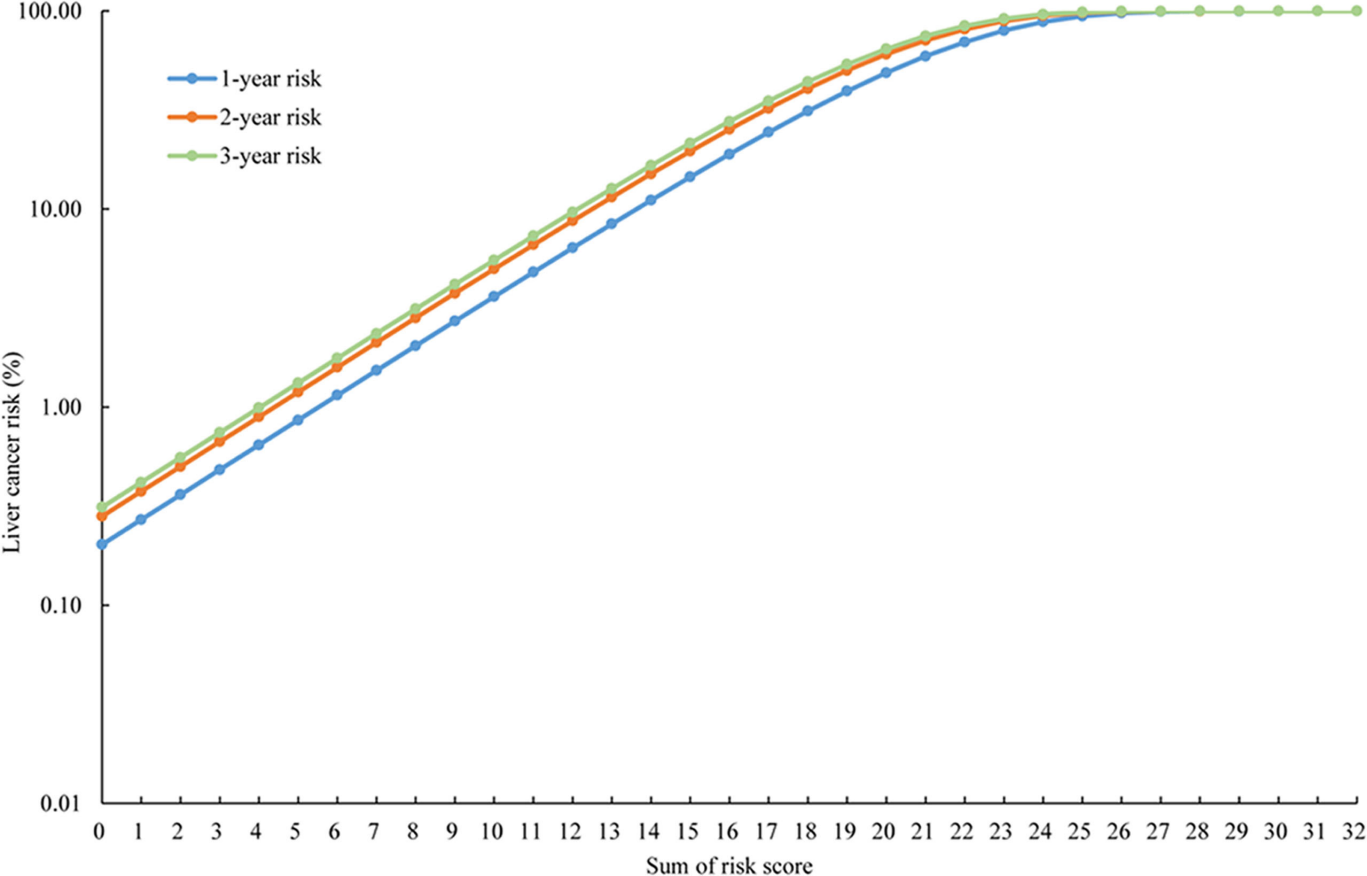
The cumulative risk of developing liver cancer of risk score at 1 year, 2 years, and 3 years.

A simple-to-use risk score was derived based on the risk prediction model, ranging from 0 to 32 (**Table 3**), which also could be used to estimate the risk of developing liver cancer roughly. For example, a male patient (risk score = 3), aged 40 years (risk score = 1), with a BMI of 30 (risk score = 0), without psychological trauma (risk score = 0), whose mother was not diagnosed with liver diseases (risk score = 0), with alcohol consumption of 600 g/week ethanol (risk score = 3) would have a cumulative risk score of 7, and the 3-year projected liver cancer risk was 1.888% (**Table 4**). Cumulative risk of developing liver cancer of risk score at 1 year, 2 years, and 3 years for each score is also shown in **Figure 2**.

**Table 3:** Components of risk score.

Variables	Category	Risk score
Sex	Female	0
	Male	3
Age	35–39	0
	40–44	1
	45–49	2
	50–54	3
	55–59	4
	60–64	5
	65–70	6
BMI	≥25	0
	<25	1
Alcohol consumption (g/week ethanol)	No	0
	<550	0
	≥550	3
Liver diseases in mothers	No	0
	Yes	2
Psychological trauma	No	0
	Yes	2

**Table 4:** Application of the risk prediction model or the risk score to specific individual.

Risk factors	Case 1	Case 2	Case 3	Case 4	Case 5
Age (years)	40	50	55	40	40
Sex	M	M	F	M	F
BMI	30	18	27	27	27
Psychological trauma	N	Y	Y	Y	Y
Liver diseases in mothers	N	Y	Y	N	N
Alcohol consumption (g/week ethanol)	600	700	0	0	0
f,M	−0.058	1.99	0.268	−0.331	−1.21
Risk score	7	14	8	6	3
3-year liver absolute risk, %	1.888	13.739	2.607	1.44	0.601

M, Male; F, Female; N, No; Y, Yes.

**Figure 3:**
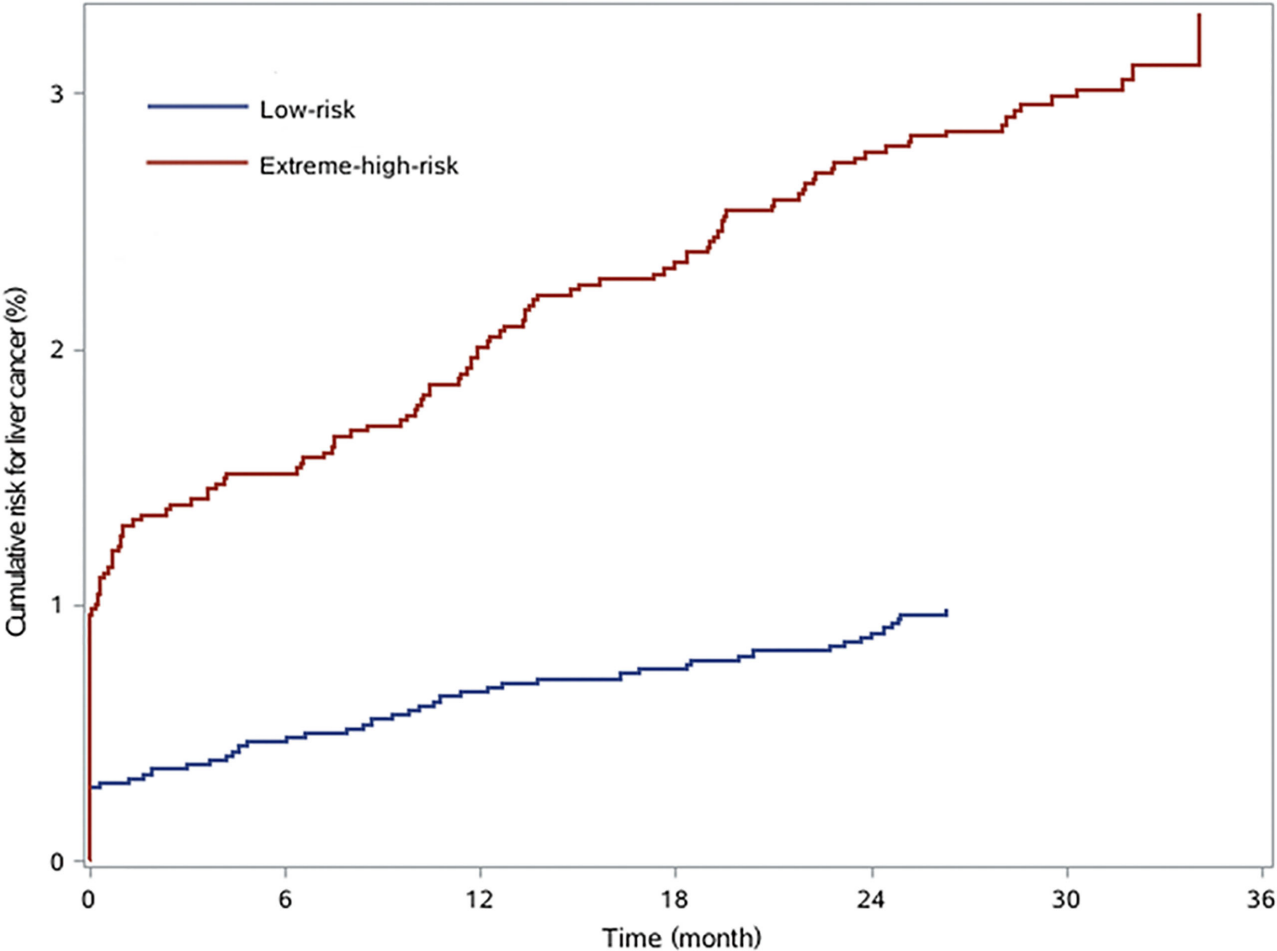
The cumulative risk for liver cancer in chronic hepatitis B patients with low risk and extreme high risk.

According to the assessment indices, the optimal cutoff value of the risk score was set at 5 with a sensitivity of 93.10%, and 76.02% of patients were considered extremely high risk. By applying the cutoff point of 5 for the risk score, 2,527 patients and 8,009 patients were divided into low-risk and extreme-high-risk groups, respectively (**Table 5**). The cumulative risk of liver cancer in the extreme-high-risk group was significantly higher than that in the low-risk group (*p* < 0.05, **Figure 3**).

**Table 5:** The accuracy of the risk score for liver cancer.

Risk scores	High-risk individuals (%)	Indices			
		Liver cancer (*N*)	Sensitivity (%)	Specificity (%)	Youden index (%)
0	10,536 (100.00)	203	100.00	0.00	0.00
1	10,490 (99.56)	203	100.00	0.45	0.45
2	10,287 (97.64)	202	99.51	2.40	1.91
3	9,882 (93.79)	201	99.01	6.31	5.32
4	9,092 (86.29)	198	97.54	13.93	11.47
5	8,009 (76.02)	189	93.10	24.32	17.42
6	6,632 (62.95)	167	82.27	37.43	19.70
7	4,897 (46.48)	148	72.91	54.04	26.95
8	3,428 (32.54)	119	58.62	67.98	26.60
9	2,239 (21.25)	94	46.31	79.24	25.55
10	1,151 (10.92)	69	33.99	89.53	23.52
11	491 (4.66)	334	16.75	95.58	12.33
12	220 (2.09)	19	9.36	98.05	7.41
13	45 (0.43)	3	1.48	99.59	1.07

### Performance of the Liver Risk Prediction Model

**Figure 4:**
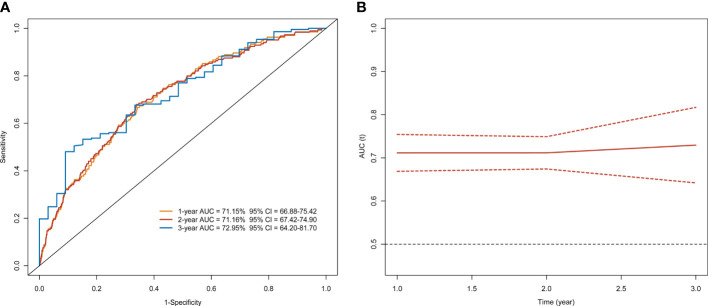
Time-dependent ROC curve and AUC analyses of prediction for developing liver cancer. **(A)** ROC and **(B)** AUC.

**Figure 5:**
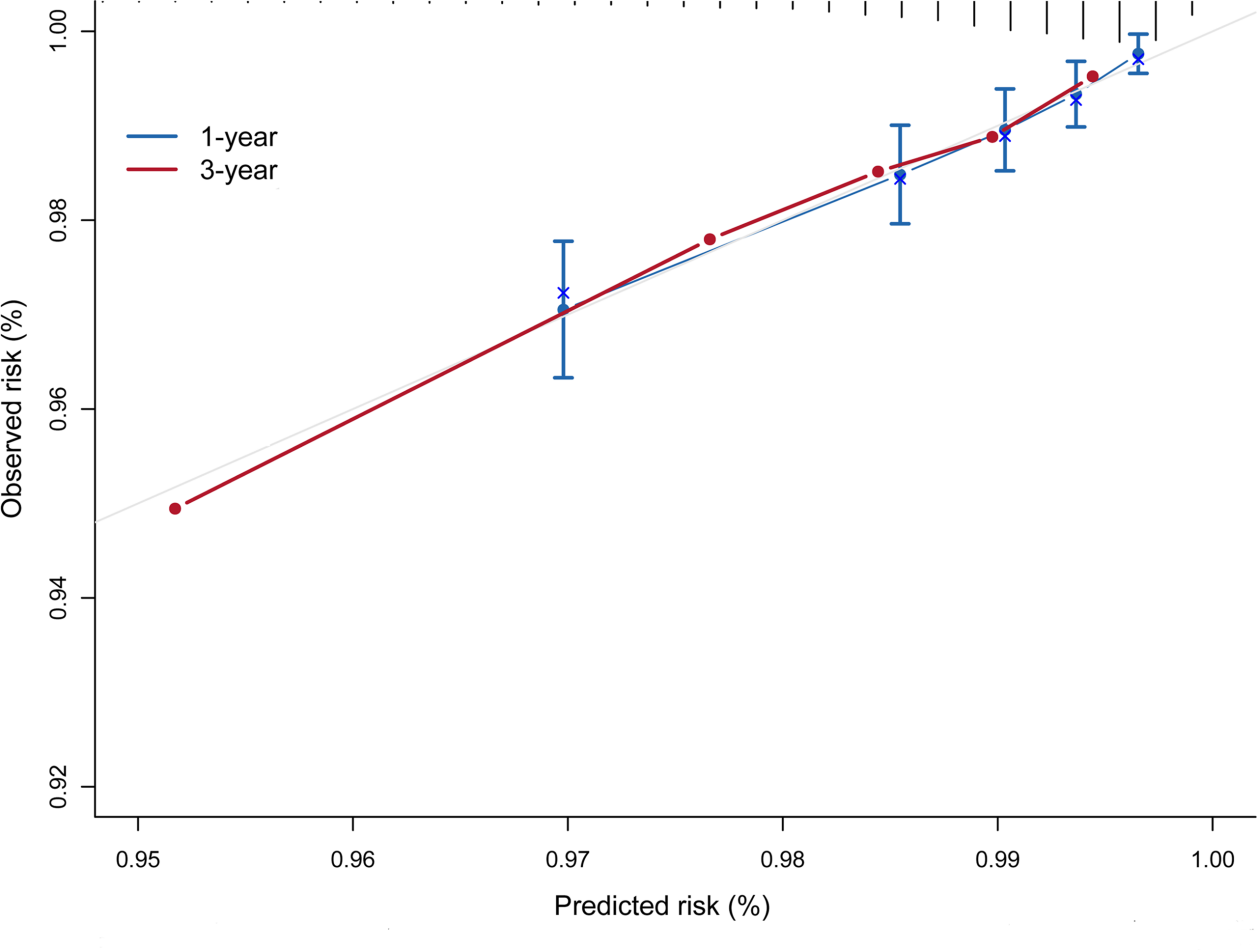
Calibration curve of the prediction model.

The risk prediction model presented a satisfactory discrimination with AUROC of 71.15% (95% CI = 66.88–75.42), 71.16% (95% CI = 67.42–74.90), and 72.95% (95% CI = 64.20–81.70) at 1 year, 2 years, and 3 years, respectively. The interval validation showed that the C-index was 0.70 (95% CI = 0.67–0.74) (**Figure 4**). Calibration curves were generated for 1 year and 3 years to evaluate the calibration of the prediction model, suggesting an excellent agreement between the observed risk and predicted probability of liver cancer (**Figure 5**).

## Discussion

This study developed and internally validated a risk prediction model and corresponding 32-point risk score for projecting the individualized absolute cumulative risk of liver cancer based on the six widely accessible and easily measured variables: age, sex, alcohol consumption, BMI, psychological trauma, and liver diseases in mothers, presenting an excellent discriminative ability and calibration. The risk scores successfully categorized HBV carriers into the extreme-high-risk and low-risk groups with significant differences in liver cancer cumulative incidence. In addition, to our knowledge, this is the first study to develop a live cancer risk prediction model only on the basis of traditional epidemiological parameters in patients chronically infected with HBV, which could be used in the primary screening stage for liver cancer.

The risk score developed in our study can provide guidance on whether HBV carriers need to receive the second-stage screening. First, according to Global Hepatitis Report 2017, more than 90 million patients are chronically infected with HBV in China at present (23). Apparently, it will cause unnecessary resource waste if all chronic hepatitis B (CHB) patients underwent regular liver cancer screening, especially for low-risk individuals. Second, further examination including AFP and ultrasound may result in psychological burden, blooding, and anxiety. Upon these considerations, the application of the risk scores at the primary screening stage would be necessary, which could narrow down the coverage of high-risk individuals of liver cancer for priority of liver cancer screening in CHB patients significantly. The results showed that CHB patients with a score of less than 5 may not need vigorous liver cancer screenings or follow-up within 3 years, and 76.02% of CHB patients were found to be at extremely high risk of our study. In our study, we aimed to identify individuals who had extremely high risks of liver cancer. When the cutoff value was set at 5, sensitivity and specificity could be relatively optimal. At the same time, we could detect as many potential extreme-high-risk patients as possible. Additionally, the individualized absolute risk of liver cancer could also be estimated at different time points, so patients chronically infected with HBV can monitor their health status by themselves. However, we have to note that external validation is warranted before applying our risk prediction model due to the diverse characteristics of the population in different regions.

Several variables are well-established risk factors associated with the development of liver cancer that have not been included in the final model. Smoking could increase the risk of liver cancer, which was statistically significant in the univariate analysis of our study; however, no significant association was observed in the multivariate analysis. Long-time follow-up is needed in the future to check the contribution of smoking to liver cancer development. Given the significant difference in the smoking rate and susceptibility of smoking between males and females, similar to the alcohol consumption (24), it is unreasonable to apply the same cutoff points to classify smoking intensity for men and women. In addition, to strengthen our results, we also classified smoking intensity for males and females with the application of the same criteria and achieved the identical conclusion. Cirrhosis as a strong risk factor of liver cancer refers to a disease in which liver cells become damaged and are replaced by scar tissue (25), irreversible in its advanced stage. To diagnose cirrhosis, you need to provide medical history, a physical exam, and a series of tests such as blood, imaging test, and liver biopsy (26). However, a standardized definition of cirrhosis has not yet been reached well so far, and thus, the diagnosis of cirrhosis is subjective to some extent. In our study, to make it user-friendly and navigate the prediction model for people accurately, we therefore did not incorporate cirrhosis into the final risk prediction model. Obesity is thought to be an independent risk factor of developing liver cancer (27). Michikawa et al. (28) devised a risk score containing age, sex, alcohol consumption, body mass index, diabetes, coffee consumption, and hepatitis B and C virus infection from 17,654 Japanese participants, including approximately 434 CHB patients and 970 patients with HCV infection. Individuals with BMI ≥ 25 had a higher risk of liver cancer, compared with people with low BMI. However, an opposite result in our study was found, which might be associated with the different population characteristics. Participants in the present study are all chronically infected with HBV for a long time, presenting a poor health status and limited liver function.

A number of risk prediction models have been developed to identify high-risk individuals of liver cancer development in HBV carriers. Yang et al. (29) developed a 17-point risk score estimating the risk of developing liver cancer at 3, 5, and 10 years on the basis of age, sex, serum alanine aminotransferase concentrations, HBV DNA levels, and HBeAg serostatus. Wong et al. (30) derived a nomogram from a cohort of 1,005 CHB patients consisting of age, albumin, bilirubin, HBV DNA, and cirrhosis. Both studies presented a satisfactory accuracy in predicting the probability of liver cancer in patients with HBV infection, but their main objective was to provide an accurate instrument in diagnosing liver cancer in clinical environment. The two risk scores may not be adaptable as a primary screening method. Noteworthy, many risk scores put too much emphasis on viral factors, limiting their widespread application. Fan et al. (31) generated aMAP score without regard to any hepatitis virus in a cohort of patients chronically infected with HBV using 4 non-viral variables, which are age, male sex, albumin–bilirubin score, and platelets counts, respectively. Although the high discriminatory performance allowed a further improvement in the detection of liver cancer, the formula for the aMAP score is relatively complex.

Our study has some notable strengths. First, the dataset used in this study was from a large cohort of patients chronically infected with hepatitis B in seven sites, avoiding selection bias effectively. We adopted the same criterion to identify liver cancer for each site and each person. All liver cancer cases diagnosed at baseline were identified further through CT or MRI. Cases collected at the follow-up stage were matched through high-quality cancer registries and death surveillance system. Furthermore, most cases were found through active follow-up such as a home visit or telephone communication. Second, the risk prediction model could quantify the 3-year absolute risk of liver cancer in CHB patients without any laboratory tests, suggesting information for epidemiological workers or clinicians to determine who should or should not need to receive further examination regarding their individual annual risk of developing liver cancer. However, we still have to acknowledge several limitations. External validation has not yet been carried out, limiting the generalizability of the risk prediction model to a certain extent. In addition, we did not consider the role of HCV to liver cancer development. It is necessary to incorporate the HCV status in the development of the risk prediction model in the future studies in view of the etiology of liver cancer in China, which will be beneficial to calculate the risk of liver cancer.

## Conclusions

A simple-to-use risk prediction model of age, sex, alcohol consumption, BMI, psychological trauma, and liver diseases in mothers was developed and internally validated, which could quantify the 3-year absolute risk of liver cancer in patients with HBV. Extreme-high-risk individuals could be identified effectively by the new scoring system. This risk prediction model could be used as a primary screening method for liver cancer.

## Data Availability Statement

The raw data supporting the conclusions of this article will be made available by the authors, without undue reservation.

## Ethics Statement

This study was approved by the Ethics Review Committee of the Cancer Hospital, Chinese Academy and Medical Sciences. The patients/participants provided their written informed consent to participate in this study. Written informed consent was obtained from the individual(s) for the publication of any potentially identifiable images or data included in this article.

## Author Contributions

Conceptualization: WC and MC. Investigation: CX, HL, DS, SH, and LL. Supervision: JP. Writing—Original Draft: MC. All authors contributed to the article and approved the submitted version.

## Funding

This study was supported by the National Natural Science Foundation of China (No. 81974492), the Sanming Project of Medicine in Shenzhen (No. SZSM201911015), and the Non-profit Central Research Institute Fund of Chinese Academy of Medical Sciences (No. 2019PT320027).

## Conflict of Interest

The authors declare that the research was conducted in the absence of any commercial or financial relationships that could be construed as a potential conflict of interest.

## Publisher’s Note

All claims expressed in this article are solely those of the authors and do not necessarily represent those of their affiliated organizations, or those of the publisher, the editors and the reviewers. Any product that may be evaluated in this article, or claim that may be made by its manufacturer, is not guaranteed or endorsed by the publisher.

## Abbreviations

WHO, World Health Organization; HBV, Hepatitis B virus; HCV, Hepatitis C virus; HBsAg, Hepatitis B surface antigen; AFP, Alpha-fetoprotein; BMI, Body mass index; HR, Hazard ratio; CI, Confidence interval; AUROC, Area under receiver operating characteristic curve; CHB, Chronic hepatitis B.
